# Enhanced Facial Rejuvenation: Biostimulatory Effects of Hylan Gel Dermal Filler DX on Collagen Synthesis and Tissue Regeneration

**DOI:** 10.1007/s00266-025-05245-5

**Published:** 2025-10-13

**Authors:** Rodolfo M. Ortiz-Flores, Alejandro Escamilla-Sanchez, Borja Cidoncha-Morcillo, Luis Mastronardi, Silvia Fontenete, Regina Garcia-Delgado

**Affiliations:** 1 BE21-Hematología e Inmunoterapia, Instituto de Investigaciones Biomédicas de Málaga y Plataforma en Nanomedicina (IBIMA Plataforma BIONAND), Málaga, Spain; 2 UGC Hematología y Hemoterapia, Hospital Universitario Virgen de la Victoria, Málaga, Spain; 3 Universidad del Salvador, Buenos Aires, Argentina; 4 BioScience GmbH, Madrid, Spain; 5 Departamento de Fisiología Humana, Histología Humana, Anatomía Patológica y Educación Físico-Deportiva, Facultad de Medicina, Universidad de Málaga, Málaga, Spain

**Keywords:** Facial rejuvenation, Regenerative medicine, Dermal fillers, Hyaluronic acid, Dextranomer, Collagen synthesis, Extracellular matrix, Cytokine modulation, Biostimulation

## Abstract

**Background:**

Facial rejuvenation involves addressing the breakdown of collagen and elastin that occurs with aging. Hyaluronic acid (HA)-based dermal fillers have become a key non-invasive treatment, known for their volumizing effects and potential regenerative benefits. This study characterizes the physicochemical and rheological properties of Hylan Gel Dermal Filler DX, a novel filler combining HA and dextranomer microspheres, and evaluates its capacity to enhance extracellular matrix regeneration.

**Methods:**

Rheological analysis was conducted to determine viscoelastic properties, including storage modulus (G'), loss modulus (G''), and tan delta across frequencies (0.1–10 Hz). In vitro experiments with normal human dermal fibroblasts (NHDFs) and monocytes (J774A.1) assessed the effects of Hylan Gel Dermal Filler DX (HA + DX), Genefill Soft Fill (HA), HarmonyCa® (HA+CaHa), Radiesse® (CaHa), and Sculptra® (PLLA). Hydration and wound healing assays were also performed. Biomarker analysis focused on collagen types I, III, IV, VI, elastin, and cytokines (TGF-β1, IL-1β, IL-6, IL-10, TNFα).

**Results:**

Rheologically, HA + DX displayed a balanced elastic and viscous profile, supporting its structural integrity and adaptability in dynamic facial areas. HA + DX demonstrated high stimulation of collagen types I, III, IV, VI, and elastin production, with enhanced fibroblast migration and wound healing at 72 hours compared to other fillers. HA + DX also modulated cytokines, increasing TGF-β1 and IL-10 while reducing TNFα and IL-1β, suggesting anti-inflammatory and regenerative properties.

**Conclusions:**

HA + DX exhibited considerable benefits in collagen stimulation, wound healing, and cytokine modulation. Its favorable rheological characteristics and regenerative potential make it a promising option for facial rejuvenation and tissue regeneration.

**No Level Assigned:**

This journal requires that authors assign a level of evidence to each submission to which Evidence-Based Medicine rankings are applicable. This excludes Review Articles, Book Reviews, and manuscripts that concern Basic Science, Animal Studies, Cadaver Studies, and Experimental Studies. For a full description of these Evidence-Based Medicine ratings, please refer to the Table of Contents or the online Instructions to Authors www.springer.com/00266

**Supplementary Information:**

The online version contains supplementary material available at 10.1007/s00266-025-05245-5.

## Introduction

Facial rejuvenation is a complex endeavor that requires addressing the multifaceted effects of aging, such as sun damage, loss of volume, and skin thinning [1]. As skin ages, the collagen fibrils that provide structural integrity fragment, and the connections between fibroblasts weaken, leading to decreased elasticity and the gradual loss of a youthful skin texture [2].

Over the past decade, dermal fillers based on hyaluronic acid (HA) have emerged as a non-invasive, effective solution for mitigating the appearance of wrinkles and skin folds. Statistics from the International Society of Aesthetic Plastic Surgery indicate that around 5.56 million non-surgical procedures using HA were performed worldwide in 2023, marking a 29.1% increase compared to the previous year [3].

These fillers function by replenishing the volume loss within the dermal extracellular matrix, a characteristic feature of aging skin, thereby providing structural support to restore a more youthful appearance [4]. Beyond their mechanical function of volume replacement, recent studies have shown that HA-based fillers might offer additional regenerative benefits [5]. Emerging evidence suggests that HA fillers may stimulate the production of procollagen and growth factors critical for skin health and rejuvenation. For instance, injections of HA-based fillers have been found to enhance the proliferation of dermal fibroblasts, improve vasculature, and increase epidermal thickness [6, 7]. One notable study evaluated the effects of various HA-based injectable dermal fillers (IDFs) on extracellular matrix components [8], showing that HA fillers can stimulate the synthesis of type I collagen in human dermal fibroblast cultures. Moreover, variations in different types of fillers demonstrated diverse impacts on other key components such as type III collagen and elastin, suggesting a broad potential for skin remodeling and improvement in texture.

Further comparative research on HA-polynucleotide (HA-PN) complex fillers versus standard HA fillers revealed that the HA-PN fillers significantly enhance cell migration and stimulate collagen synthesis in both human and mouse fibroblasts. This biostimulatory effect suggests the potential of HA-PN fillers not only for restoring volume but also for promoting skin regeneration and volumization [9]. Such results align with earlier findings that HA-based fillers support the in vitro production of type I collagen, underscoring their capacity for long-term structural improvement in aged skin [10]. In this context, the present study seeks to explore a new generation of dermal fillers that combine HA with dextranomer microspheres. Dextranomer, a biodegradable hydrophilic glucose polymer, is composed of cross-linked dextran molecules. Produced by lactic acid bacteria such as Leuconostoc mesenteroides and *Streptococcus mutans* [11] dextranomer microspheres have shown promising results when combined with non-animal stabilized hyaluronic acid, particularly in medical applications such as fecal incontinence treatments [12, 13]. In facial aesthetics, dextranomer fillers have demonstrated a favorable safety profile and efficacy in augmenting facial tissue defects [14].

The objective of this study is to characterize the physicochemical and rheological properties, as well as evaluate the cellular and molecular effects, of a novel dermal filler composed of hyaluronic acid (HA) and dextranomer microspheres (Hylan Gel Dermal Filler DX) on extracellular matrix regeneration. The study also aims to compare the biostimulatory effects of HA + DX with other commercially available dermal fillers, focusing on collagen and elastin production, fibroblast migration, cytokine modulation, and hydration, to assess its potential for facial rejuvenation and tissue regeneration. The findings from this study offer valuable insights into the biostimulatory properties of this hybrid filler, highlighting its potential as an innovative option for non-surgical facial rejuvenation.

## Materials and Methods

### Study Objective

To evaluate the biostimulatory effects of Hylan Gel Dermal Filler DX and other commercially available HA-based fillers, Genefill Soft Fill (HA), HarmonyCa® (HA + CaHa), Radiesse® (CaHa), and Sculptra® (PLLA), *in vitro* experiments were conducted using normal human dermal fibroblasts (NHDFs) and monocytes (J774A.1). The aim was to assess their impact on the production of extracellular matrix (ECM) components (type I, II, III, and VI collagen, elastin, and transforming growth factor [TGF]-β1) as well as pro- and anti-inflammatory cytokines (interleukin [IL]-1β, IL-6, IL-10, and tumor necrosis factor [TNF]α).

### Preparation of Filler Extracts

Each dermal filler (0.2 g or 1 g) was dissolved in 1 mL of extraction medium composed of Dulbecco’s Modified Eagle’s Medium (DMEM) supplemented with 10% fetal bovine serum (FBS), 1% antibiotic-antimycotic solution (penicillin, streptomycin, amphotericin B), and 1% non-essential amino acids.

- Sculptra® (PLLA): Reconstituted with 1 mL of sterile water per vial (final concentration: 367.5 mg/mL), and 0.544 mL of this solution was mixed with 0.456 mL of the same culture medium (MEM-based), yielding a final concentration of 0.2 g/mL.
- Radiesse® (CaHa): With a concentration of 550 mg/mL in 1.5 mL prefilled syringes, 0.364 mL was diluted in 1 mL of culture medium. The mixture was vortexed to ensure a homogeneous suspension.

### Hyaluronic Acid Filler Characteristics

Hylan Gel Dermal Filler DX consists of cross-linked hyaluronic acid sodium salt (14.0 mg/mL), dextranomer (DEAE Sephadex; 50.0 mg/mL), and sodium chloride (6.9 mg), in water for injection (1.0 mL total volume). The filler has a viscosity of 39,000–51,000 mPas, molecular weight of 750 kDa, and particle size between 80–150 µm. It is indicated for volume restoration and facial tissue augmentation.

### Physicochemical Characterization

The pH of each sample was determined in duplicate using a 913 Metronome pH meter for accuracy. To evaluate the swelling factor (SwF), 1.5 mL of 1x PBS was introduced to the filler sample, which was then incubated for 24 hours at ambient conditions (23°C and 77% relative humidity). Following incubation, the PBS was carefully extracted, and the remaining filler volume was quantified using an automated electronic pipette. Clarity assessments were carried out in duplicate using the BYK-Gardner haze-gard, with narrow-angle scattering measurements. The filler sample was placed in front of the light source, and the scattering behavior was captured using sensors positioned within the light trap zone. Osmolality readings were obtained in duplicate using a standard osmometer (model 2020; Advanced Instruments, Inc., Norwood, MA).

### Rheological Properties

The rheological profile of Hylan Gel Dermal Filler DX was analyzed using a Discovery Hybrid Rheometer (DHR) with a 60 mm parallel plate and Peltier plate at 25°C. Testing was conducted at 2% strain, across frequencies from 0.1 to 4 Hz (5 data points per frequency decade), to obtain storage modulus (G′), loss modulus (G″), and tan δ. The complex modulus (G*) was calculated as:

 G^* = \sqrt{(G')^2 + (G'')^2}

Validation was achieved by calculating the coefficient of variation (CV%) across four control samples tested in parallel.

#### Fibroblast and Macrophage Cultures

Fibroblasts, NHDFs (ATCC® PCS-201-012™), were cultured in T75 flasks and maintained at 37°C with 5% CO_2_. For experimentation, cells were seeded into 24-well plates until 80% confluence, then exposed to either the extraction medium alone (control) or one of the filler extracts (0.2 g/mL in DMEM + 10% FBS, 1% antibiotics, 1% NEAA). Macrophages, J774A.1 monocytes (ATCC® TIB-67™), were cultured under identical conditions and treated similarly in 24-well plates, with exposure at 24, 48, and 72 hours.

### Co-Culture System

A Transwell system in 24-well plates was employed to investigate paracrine interactions. Fibroblasts (50,000 cells/well) were seeded in the lower chamber and allowed to attach for 24 hours. Macrophages (100,000 cells/well) were then seeded in the upper inserts with DMEM + 15% FBS and 1% antibiotics. After 24 hours, both cell types were treated with filler extracts (1 mg/mL) for an additional 24 hours. Supernatants from both chambers were harvested post-treatment, centrifuged (5000×g, 15 min, 4°C), and stored at − 70°C.

### Biomarker Quantification

At 24, 48, and 72 hours, supernatants from fibroblast, macrophage, and co-cultures were collected, centrifuged (5000×g, 15 min, 4°C), and stored at −70°C. ELISAs (Assay Genie®) were conducted in triplicate to quantify: type I, II, III, and VI collagen; elastin; TGF-β1; IL-1β, IL-6, IL-10; and TNFα, according to the manufacturer's instructions.

#### Comparative Hydration Analysis

The hydration effect of Hylan Gel Dermal Filler DX will be evaluated by assessing its capacity to retain water in the fibroblast cultures. A comparative analysis will be performed using Hylan Gel Dermal Filler DX (1 mg/mL) and commercial HA-based fillers. The ability of each filler to enhance hydration will be quantified by measuring the water retention capacity in treated NHDF cultures, with a focus on comparing their effects on cellular hydration and volume restoration. Fibroblasts were seeded at a density of 150,000 cells per well in a 6-well plate and incubated. After 24 hours of incubation, the wells were treated with the medium alone (control) or HA-based fillers (1 mg/mL). Each treatment was applied for 24, 48, and 72 hours. To evaluate the hydrating effect of each treatment, a modified water content assay was performed. Cells were harvested by washing with phosphate-buffered saline (PBS), followed by centrifugation at 2000×g, to obtain a pellet. The pellet was weighed to determine the wet weight. The samples were then dried at 60°C for 24-48 hours until a constant weight was achieved to measure the dry weight. Water content (%) was calculated using the following formula:

Water Content (%) = ((Wet Weight − Dry Weight) / Wet Weight) × 100

#### Wound Healing Assay

Scratch assays were performed on confluent NHDF monolayers in 6-well plates (150,000 cells/well). A scratch was made using a sterile pipette tip, and cells were then treated with filler extracts (1 mg/mL) or control medium. Cell migration was observed at 24, 48, and 72 hours using an AE21 inverted microscope (Motic Microscopy Inc., San Antonio, USA). Images were normalized using XnConvert and analyzed in ImageJ (v1.51i, NIH). Wound closure was quantified as a percentage of the original wound area.

#### Statistical Analysis

Data are expressed as mean ± standard error of the mean (SEM). Statistical comparisons were performed using one-way ANOVA with Tukey’s post-hoc test (GraphPad Prism 6). All experiments were conducted in triplicate. Significance thresholds were set as follows: *p *< 0.05 (*), *p *< 0.001 (**), *p *< 0.0001 (***).

## Results

### Physicochemical Characteristics of Hybrid Filler

A physicochemical analysis of Hylan Gel Dermal Filler DX was performed (Table 1). HA + DX filler presented a pH of 6.74, indicating compatibility with physiological skin conditions. The clarity of the product was measured using a light scattering assay, which revealed a turbidity level of 23%, indicating the presence of a non-transparent and opalescent formulation. The swelling factor (SwF) was observed to be 121%, indicating the product's ability to retain moisture, and the osmolality was recorded at 426 mOsmol/kg, which is within a range suitable for maintaining physiological balance in dermal tissues. These results suggest that the product possesses physicochemical properties conducive to dermal applications.

**Table 1:** Physicochemical properties of studied fillers (mean values for duplicate measurements)

pH	Clarity (%)	SwF (%)	mOsmol per kilo
6.74	23	121	426

*SwF* swelling factor

### Rheological Characteristics of Hybrid Filler

A rheological analysis was conducted on the Hylan Gel Dermal Filler DX, with the findings detailed in Table 2. The viscoelastic properties of the HA + DX filler were assessed across multiple frequencies (10 Hz, 4 Hz, 1.6 Hz, 1 Hz, and 0.1 Hz). The analysis measured key parameters such as the storage modulus (G'), loss modulus (G''), complex modulus (G*), and tan delta value at each specified frequency.

**Table 2:** Rheological properties of the HA + DX filler at varying frequencies

Frequency (Hz)	G'	G''	G*	Tan delta
10	4206,3	632,4	4253,6	0,2
4	3862	663,8	3918,7	0,2
1	3335,3	696	3407,2	0,2
0,1	2583,6	5647	2644,6	0,2

The table presents the storage modulus (G'), loss modulus (G''), complex modulus (G*), and tan delta values at frequencies of 10 Hz, 4 Hz, 1 Hz, and 0.1 Hz.

At higher frequencies (10 Hz and 4 Hz), HA + DX filler demonstrated a higher storage modulus, indicating a predominantly elastic behavior with values of 4206.3 Pa and 3862.0 Pa, respectively. The G'' was also observed to be relatively high in these frequencies, contributing to a consistent tan delta of 0.2, which suggests a stable viscoelastic profile.

At a lower frequency (1.6 Hz), there was a marked decrease in G' (33.8 Pa) and G'' (42.2 Pa), resulting in a tan delta of 1.2, indicating a shift towards a more viscous behavior. As the frequency further decreased to 1 Hz and 0.1 Hz, the G' values remained lower compared to higher frequencies, but still showed relatively elastic characteristics with tan delta values reverting to 0.2. These results indicate that HA + DX filler demonstrates a dynamic viscoelastic profile, with predominantly elastic properties at higher frequencies and increased viscous behavior at lower frequencies.

### Comparison of Water Content in Treated Cells

The hydration effect of an HA filler (containing 20 mg of crosslinked HA and 2 mg of non-crosslinked HA) and the HA + DX filler under investigation was evaluated in NHDF cells and J774A.1 monocytes at 24, 48, and 72 hours. Table 3 presents the water content percentage under each experimental condition.

**Table 3:** Water content in NHDF fibroblasts and J774A.1 monocytes treated with HA filler and HA + DX filler

Cell type	Time (hours)	Control	HA + DX	HA
NHDF	24	37,0%	41,0%^***^	49,9%^###^
	48	35,8%	38,8%^***^	42,1%^###^
	72	33,2%	37,0%^**^	34,0%
J774A.1	24	36,0%	38,0%^**^	42,0%^###^
	48	34,5%	37,0%^**^	38,7%^##^
	72	33,8%	35,0%	34,7%

Significant differences from the control (DMEM) are indicated with asterisks (*) for HA + DX filler, where **p < 0.01, and ***p < 0.001; and with hash (#) for HAfiller, where ^##^*p *< 0.01, and ^###^*p *< 0.001.

The data indicate that HA consistently provides increased water content in both NHDF cells and J774A.1 monocytes at all time points. However, this effect is more pronounced at 24 hours, and in fibroblasts. At 24 hours, HA filler achieved a significant increase in water content (49,9% in NHDF cells and 42,0% in J774A.1 monocytes) compared to control and HA + DX filler. Over time, the hydrating effect of HA filler remains superior to that of HA + DX filler, although there are no longer significant differences at 72 hours. HA filler demonstrates an exceptional capacity to enhance cellular hydration compared to HA + DX filler, particularly in the early stages, making it ideal for treatments requiring rapid hydration. However, over time, the hydration levels associated with HA + DX filler become more stable and sustained, suggesting that HA + DX filler may be a better option for long-term skin health and maintenance, offering enduring hydration benefits in addition to other regenerative properties.

### Comparison of Cell Migration Between HA Filler and HA + DX Filler

To investigate the effects of HA and HA + DX fillers on NHDF cells proliferation, a wound-healing assay was performed. Cells were treated with HA and with HA + DX fillers as culture medium only, the untreated control. “Wounds” or tears were created in the cell monolayers and incubated in a medium with the above treatments at 37°C in a 5% CO2 incubator for 0, 24, 48 and 72 hours.

**Fig. 1:**
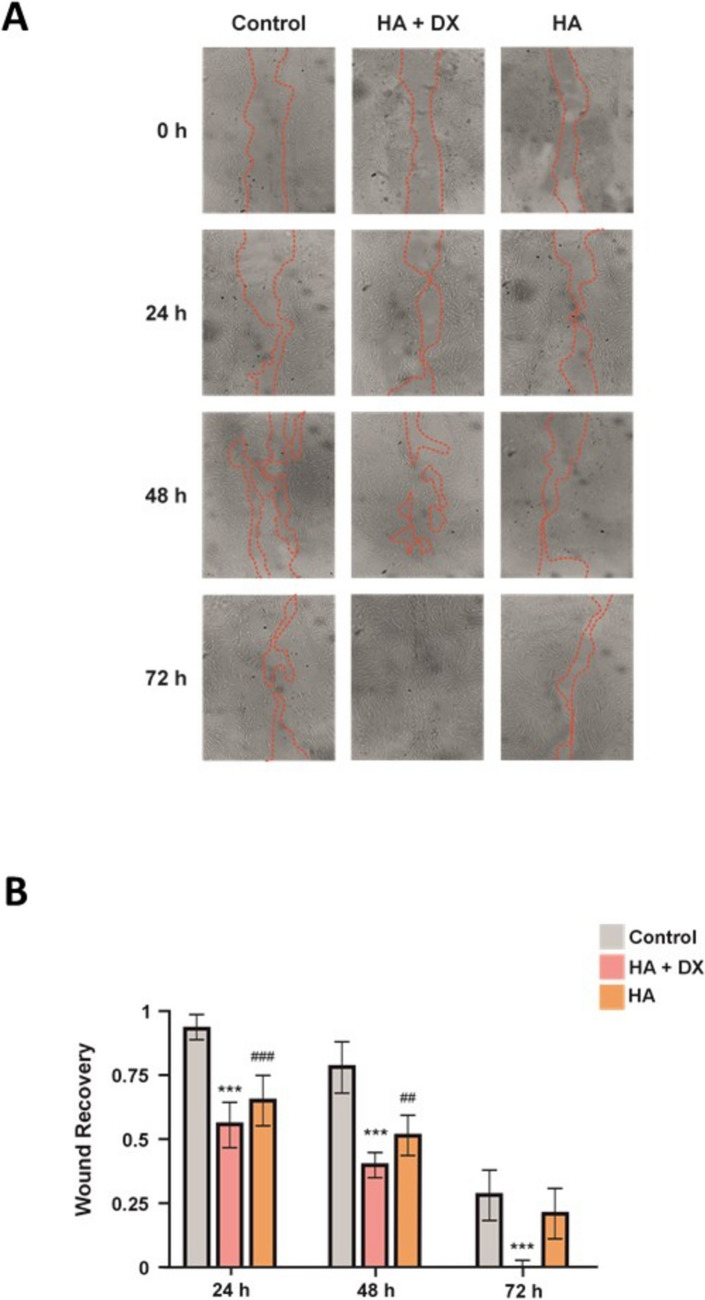
Wound healing comparison between HA + DX and HA fillers. **A** Wound healing assay on NHDF cells treated with HA + DX and HA fillers, or untreated (control, DMEM). Images were taken at 0, 24, 48, and 72 hours. At 24 hours, both fillers significantly improved wound closure compared to control (area within the red dotted line), with no differences between treatments. By 48 hours, HA-DX filler showed greater closure than HA filler. At 72 hours, HA + DX filler achieved complete closure, while HA filler showed slower progress but remained significantly better than control. **B** Quantification of wound closure rates. HA + DX filler demonstrated faster and more complete wound closure. Data shown as mean ± SEM; significant differences from the control are indicated with asterisks (*) for HA + DX filler, where ^***^p < 0.001; and with hash (#) for HA filler, where ^##^*p *< 0.01, and ^###^*p *< 0.001.

At 24 hours, significant differences in wound closure were observed between HA + DX filler and the control, and between HA filler and the control. However, neither treatment showed a significant difference compared to the other, which showed cell migration and the beginning of wound closure (Fig. 1A). At 48 hours, HA + DX filler showed greater efficacy in wound closure compared to HA filler. Wounds treated with HA + DX filler showed a more advanced closure, while HA filler continued to show a slower closure rate, although still significant compared to the control (Fig. 1A). At 72 hours, HA + DX filler managed to close the wound completely, evidencing a high stimulation capacity for cell migration and proliferation. On the other hand, HA filler continued to show a less efficient wound closure compared to HA + DX filler, although significantly superior to the control (Fig. 1A).

These results suggest that, although both treatments are effective in improving wound healing in NHDF cells compared to the control, HA + DX filler has a significantly superior capacity to accelerate the wound closure process, achieving complete closure at 72 hours, compared to HA filler, which shows a more gradual effect on wound healing (Fig. 1B).

### Collagen and Elastin Production in Fibroblasts Cells

To assess the impact of HA filler and HA + DX filler on collagen (COL) and elastin production in NHDF cells, the levels of COL-I, COL-III, COL-IV, COL-VI, and elastin were measured at 24, 48, and 72 hours of incubation. The results revealed distinct effects of both treatments over time.

**Fig. 2:**
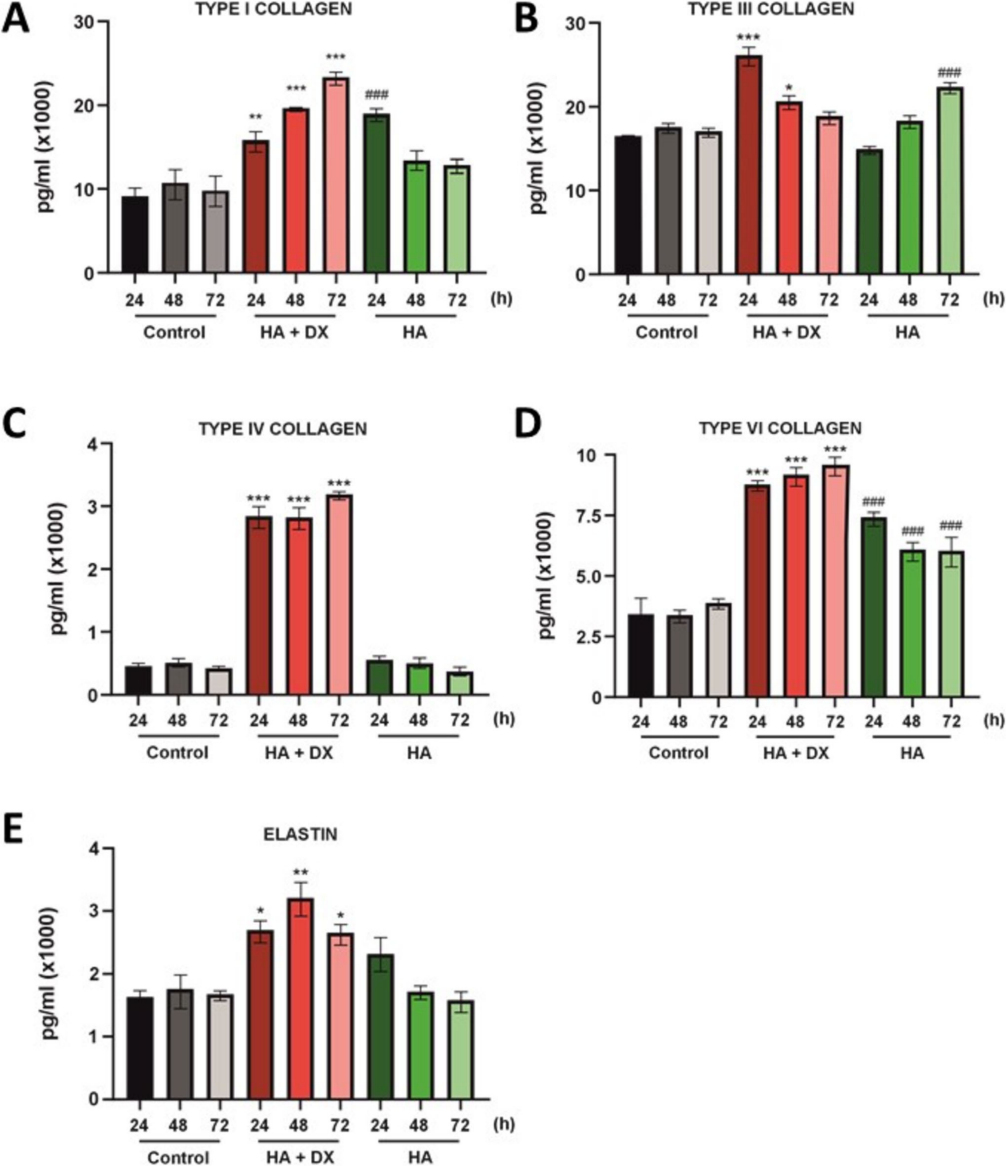
Collagen and elastin production in NHDF cells treated with HA + DX and HA filler. **A**–**E** Measurement of COL-I, COL-III, COL-IV, COL-VI, and elastin levels at 24, 48, and 72 hours after treatment with HA + DX and HA filler, and untreated control. HA + DX filler significantly increased COL-I, COL-III, COL-IV, COL-VI, and elastin production at all time points, with the highest effects observed at 24 hours. HA filler showed a moderate increase in COL-I and COL-III at 24 and 72 hours, respectively, but had limited effects on COL-IV and elastin production. Data shown as mean ± SEM; significant differences from the control are indicated with asterisks (*) for HA + DX filler, where ^*^p < 0.05, ^**^p < 0.01, and ***p <  0.001; and with hash (#) for HA filler, where ^###^*p *< 0.001.

For COL-I production, HA + DX significantly increased levels at all time points (24, 48, and 72 hours) compared to the control, with the highest increase observed after 24 hours (Fig. 2A). In contrast, HA filler showed a less pronounced effect, with a significant increase at 24 hours but returning to control levels by 48 and 72 hours. Regarding COL-III production, cells treated with HA + DX exhibited a significant increase at 24 hours compared to the control (Fig. 2B), though this effect diminished beyond 48 hours. HA filler, on the other hand, only induced a significant increase in COL-III at 72 hours, without notable effects at earlier time points.

In terms of COL-IV production, HA + DX stimulated a sustained increase in COL-IV levels up to 72 hours, showing a consistent and significant effect compared to the control. In contrast, HA filler did not significantly affect COL-IV production throughout the incubation period (Fig. 2C). When evaluating COL-VI, both treatments led to increased production at 24, 48, and 72 hours (Fig. 2D). However, the increase was more pronounced and sustained with HA + DX, while HA produced a lower, though still significant, increase compared to the control.

For elastin production, HA + DX significantly promoted elastin synthesis in NHDF cells starting at 24 hours compared to the control (Fig. 2E), maintaining this effect over time. In contrast, HA filler did not induce significant elastin production at any of the time points measured. These findings highlight the superior ability of HA + DX to stimulate the production of various collagen types and elastin in fibroblasts, while HA filler shows more limited and time-dependent effects, particularly in its ability to stimulate COL-I, COL-III, and COL-VI production.

### Cytokine Production in Fibroblasts Cells

**Fig. 3:**
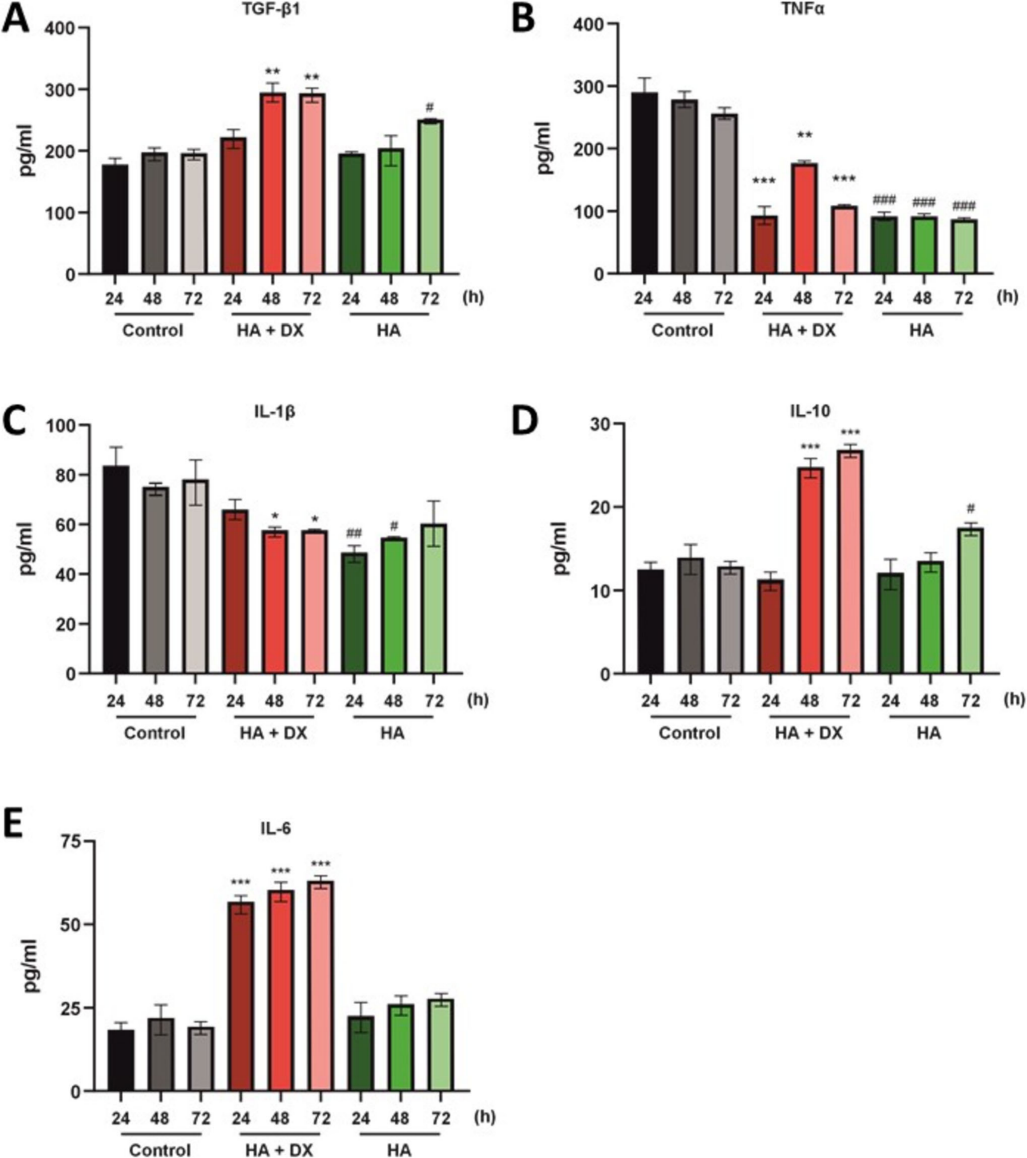
Cytokine production in NHDF cells treated with HA + DX filler and HA filler. **A**–**E** Cytokine levels (TGF-β1, TNFα, IL-1β, IL-10, and IL-6) measured in NHDF cells after treatment with HA + DX filler, HA filler, and untreated control at 24, 48, and 72 hours. HA + DX filler significantly increased TGF-β1, IL-10, and IL-6 production at 48 and 72 hours, while reducing IL-1β levels. HA filler showed a less pronounced cytokine response, primarily affecting TNFα and IL-10 at 72 hours. Data shown as mean ± SEM; significant differences from the control are indicated with asterisks (*) for HA + DX filler, where ^*^p < 0.05, ^**^p < 0.01, and ^***^p < 0.001; and with hash (#) for HA filler, where ^#^*p *< 0.05, ^##^*p *< 0.01, and ^###^*p *< 0.001.

To assess the effect of HA + DX and HA fillers on cytokine production in NHDF cells, levels of TGF-β1, TNFα, IL-1β, IL-10, and IL-6 were measured at 24, 48, and 72 hours of incubation. HA + DX treatment resulted in a significant increase in TGF-β1 production in NHDF cells at 48 and 72 hours compared to the control (Fig. 3A). HA filler also elevated TGF-β1 levels, but this increase was less pronounced, with a significant rise only observed at 72 hours compared to the control. TNFα levels showed significant changes in fibroblasts treated with both HA + DX and HA filler at all time points evaluated (Fig. 3B). IL-1β production was significantly lower in HA + DX -treated fibroblasts at 48 and 72 hours compared to the control (Fig. 3C). Treatment with HA filler also caused significant changes in IL-1β production at earlier time points, though IL-1β levels appeared to normalize at 72 hours compared to the control. HA + DX induced a significant increase in IL-10 levels at 48 and 72 hours compared to the control (Fig. 3D), while HA filler significantly increased IL-10 production only at 72 hours compared to the control. Finally, incubation with HA + DX led to a noticeable and significant increase in IL-6 levels in NHDF cells at 48 and 72 hours compared to the control (Fig. 3E). In contrast, treatment with HA filler did not affect IL-6 production compared to the control.

These findings suggest that HA + DX more effectively modulates cytokine production in NHDF cells, particularly by significantly increasing TGF-β1, IL-10, and IL-6 levels while reducing IL-1β. This modulation of cytokine profiles could have significant implications for collagen production and fibroblast activity, highlighting HA + DX’s potential in enhancing regenerative and therapeutic applications.

### Cytokine Production In Monocytes Cells

**Fig. 4:**
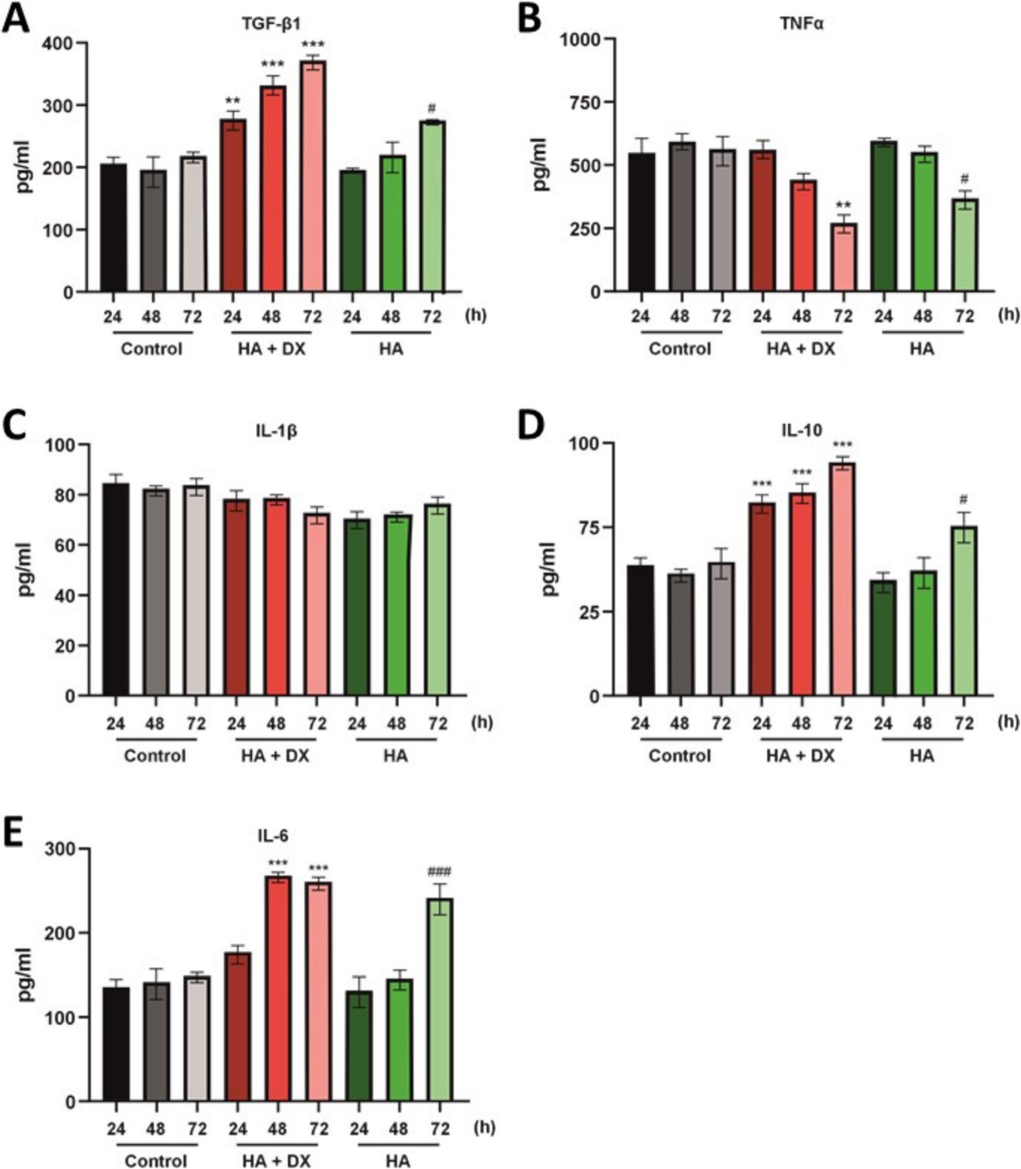
Cytokine production in J774A.1 monocytes treated with HA + DX filler and HA filler. **A**–**E** Cytokine levels (TGF-β1, TNFα, IL-1β, IL-10, and IL-6) measured in J774A.1 monocytes following treatment with HA + DX filler, HA filler, and untreated control at 24, 48, and 72 hours. HA + DX filler significantly increased TGF-β1, IL-10, and IL-6 production, while reducing TNFα at 72 hours. HA filler also affected cytokine levels but to a lesser extent, primarily influencing TGF-β1 and IL-6 at later time points. Data shown as mean ± SEM; significant differences from the control are indicated with asterisks (*) for HA + DX filler, where ^**^p < 0.01, and ^***^p < 0.001; and with hash (#) for HA filler, where ^#^*p *< 0.05, and ^###^*p *< 0.001.

Cytokine production in J774A.1 monocytes treated with HA + DX and HA fillers was evaluated at 24, 48, and 72 hours. Treatment with HA + DX resulted in a significant increase in TGF-β1 production starting at 24 hours, compared to the control (Fig. 4A). HA filler also elevated TGF-β1 levels, though this effect was less pronounced and observed only at 72 hours. HA + DX led to a significant reduction in TNFα production at 72 hours compared to the control (Fig. 4B), while HA filler also reduced TNFα levels, albeit to a lesser extent, at the same time point. Production of IL-1β did not exhibit significant changes in J774A.1 monocytes treated with either HA + DX or HA filler across all time points evaluated (Fig. 4C). Conversely, HA + DX induced a substantial increase in IL-10 production starting at 24 hours compared to the control (Fig. 4D), with HA filler also significantly increasing IL-10 levels, though to a lesser extent, at 72 hours. Additionally, IL-6 production showed a significant increase with HA + DX treatment at 48 and 72 hours compared to the control (Fig. 4E). HA filler also significantly elevated IL-6 levels, but this effect was less marked and observed only at 72 hours.

These findings indicate that HA + DX has a more pronounced effect on stimulating TGF-β1, IL-10, and IL-6 production while also reducing TNFα in J774A.1 monocytes compared to HA filler. The differential effects on cytokine profiles suggest that HA + DX might be more effective in modulating inflammatory responses and influencing the extracellular matrix compared to HA filler, potentially offering greater therapeutic benefits. Interesting, TGF-β1 is a key cytokine known for its role in stimulating collagen production by promoting fibroblast proliferation and collagen synthesis, thereby supporting the remodeling and repair of extracellular matrix.

### Hydration Effect Comparison of HA + DX Filler with Fillers

The hydration effects of HA + DX filler and other commercial fillers (Harmonyca® (HA + CaHa), Radiesse® (CaHa) and Sculptra® (PLLA)) were evaluated in NHDF cells and J774A.1 monocytes over 24, 48, and 72 hours. Table 4 presents the percentage of water content in the cells under each experimental condition.

**Table 4:** Water content in NHDF fibroblasts and J774A.1 monocytes treated with HA + DX, HA + CaHa, CaHa, and PLLA

Cell type	Time (hours)	Control	HA + DX	HA + CaHa	CaHa	PLLA
NHDF	24	37,2%	39,0%^**^	38,5%^##^	38,8%^&&^	36,5%^†^
	48	36,0%	37,1%^*^	38,0%^##^	38,1%^&&^	36,2%
	72	34,6%	35,7%^*^	37,5%^##^	37,9%^&&^	35,8%
J774A.1	24	37,0%	40,0%^***^	41,0%^###^	41,0%^&&&^	38,0%^†^
	48	36,8%	38,1%^*^	37,8%^#^	39,5%^&&&^	37,5%
	72	35,8%	36,7%^*^	37,1%^#^	37,1%^&&^	36,2%

Significant differences from the control (DMEM) are indicated with asterisks (*) for HA + DX, where *p  < 0.05, **p < 0.01, and ***p < 0.001; with hash (#) for HA + CaHa, where ^#^*p *< 0.05, ^##^*p *< 0.01, and ^###^*p *< 0.001; with et (&) for CaHa, where ^&&^*p *< 0.01, and ^&&&^*p *< 0.001, and with dagger (†) for PLLA, where ^†^*p *< 0.05.

The data indicate that all four treatments— HA + DX filler, HA + CaHa, CaHa, and PLLA—enhance cellular hydration compared to the control (DMEM) across all time points. CaHa consistently provided high water content in both NHDF cells and J774A.1 monocytes, particularly at 24 hours, achieving hydration levels of 38.8% and 41.0%, respectively. HA + CaHa also demonstrated strong hydration effects, with values slightly lower than CaHa but higher than HA + DX filler and PLLA. HA + DX filler showed a consistent but moderate hydration effect, with improvements over the control, though generally lower than HA + CaHa and CaHa. PLLA, while effective, displayed the lowest hydration levels among the four treatments, although it still provided a significant increase compared to the control. HA + CaHa and CaHa exhibit superior hydration capabilities compared to HA + DX filler and PLLA, particularly in the early stages (24 hours). However, HA + DX filler remains a competitive option, maintaining hydration over time and potentially offering additional benefits beyond hydration. PLLA, while effective, lags behind the other treatments in terms of hydration, especially at earlier time points.

### Comparison of Cell Migration among Dermal Fillers

To assess the impact of different treatments on cell migration, we compared the effects of HA + DX, and HA + CaHa (and CaHa, and PLLA) on fibroblast movement, a critical aspect of tissue repair and regeneration. Cell migration plays a pivotal role in wound healing and tissue remodeling, making it a key parameter to assess the efficacy of regenerative treatments.

**Fig. 5:**
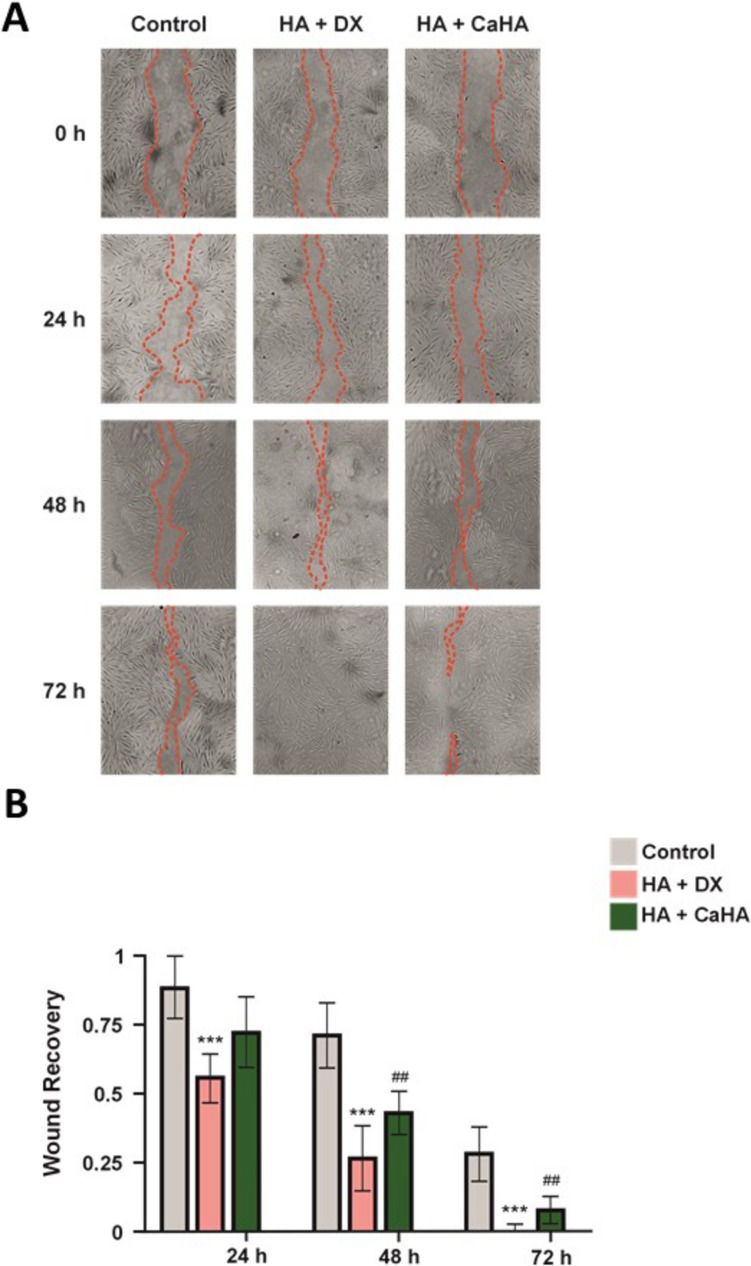
Comparison of cell migration between HA + DX and HA + CaHa fillers**.** **A** Microscopic images illustrating fibroblast migration after 24, 48 and 72 hours of treatment with control, HA + DX filler and HA + CaHa filler. HA + DX filler demonstrated the highest level of migration, followed by HA + CaHa. **B** Quantitative analysis of cell migration across the treatments. HA + DX filler significantly enhanced fibroblast migration compared to HA + CaHa filler indicating its superior efficacy in promoting cell movement critical for tissue repair. Data are presented as mean ± SEM; significant differences from the control are indicated with asterisks (*) for HA + DX filler, where ^***^p < 0.001 and with hash (#) for HA + CaHa, where ^##^*p *< 0.01.

HA + DX demonstrated the most significant enhancement of fibroblast migration compared to HA + CaHa. At all time points assessed, HA + DX substantially increased cell migration compared to the control group and HA + CaHa (Fig. 5A). The significant effect of HA + DX on cell migration is also observed when compared to CaHa and PLLA treatments (Supplementary Fig. 1). Statistical analysis further supported this finding as HA + DX-induced migration was significantly higher (*p* < 0.01) compared to HA + CaHa (Fig. 5B) and to the other products (Supplementary 1). This enhanced migration is likely driven by HA + DX’s potent stimulation of cell signaling pathways involved in migration and extracellular matrix remodeling, ultimately promoting more effective tissue regeneration. HA + CaHa also exhibited a strong effect on fibroblast migration, although slightly less pronounced than HA + DX. HA + CaHa significantly enhanced cell movement compared to control and the remaining treatments. These effects suggest a synergistic role of HA + CaHa in enhancing fibroblast migration, thereby favoring tissue repair. CaHa and PLLA showed comparatively minor effects on fibroblast migration. CaHa promoted a moderate increase in cell migration, but it was less significant compared to HA + DX and HA + CaHa. PLLA exhibited the least enhancement of fibroblast movement, although it still demonstrated some positive impact.

In summary, these results highlight HA + DX as the most effective treatment for promoting fibroblast migration, followed by HA + CaHa. CaHa and PLLA were less effective, with PLLA showing the least effect (or the slowest effect). These findings suggest that HA + DX might offer superior tissue regeneration and repair benefits in clinical settings where enhanced fibroblast migration is critical.

### Comparison of Collagen and TGF-β1 Production in the Co-Culture System

HA + DX filler has demonstrated a robust ability to stimulate collagen production and other key components of the extracellular matrix in NHDF cells at 24 hours. The results were compared with an untreated control and three commercial inducers: HA + CaHa (and CaHa and PLLA).

#### Production of Extracellular Matrix Components

**Fig. 6:**
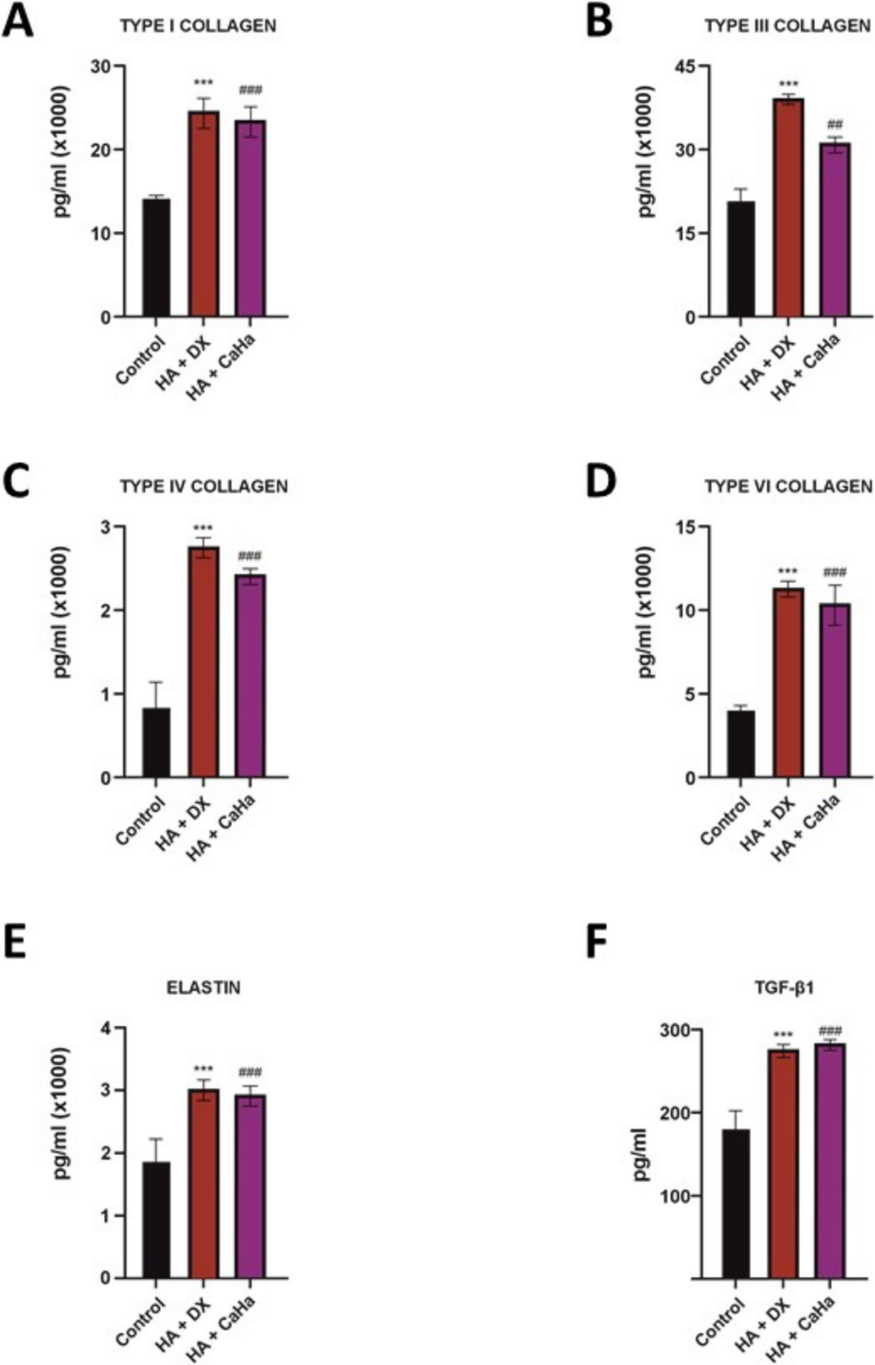
Comparison of collagen and TGF-β1 production in a co-culture system between HA + DX and HA + CaHa fillers**.** **A**–**F** Levels of collagen (COL-I, COL-III, COL-IV, COL-VI), elastin, and TGF-β1 measured in fibroblast supernatants after 24-hour treatment with an untreated control, HA + DX filler, HA + CaHa filler. HA + DX filler significantly increased the production of all evaluated collagen types, elastin, and TGF-β1. HA + CaHa filler demonstrated notable effects, particularly on COL-I, COL-III, and TGF-β1. Data are presented as mean ± SEM; significant differences from the control are indicated with asterisks (*) for HA + DX filler, where ^***^p < 0.001 and with hash (#) for HA + CaHa filler, where ^##^*p *< 0.01, and ^###^*p *< 0.001.

In the co-culture performed using the transwell system, which allowed for the analysis of the fibroblast supernatant after 24 hours, significant differences in the production of collagen (COL-I, COL-III, COL-IV, COL-VI), elastin, and TGF-β1 were observed across the various treatments, as shown in Fig. 6. In the case of the HA + DX filler treatment, a significant increase was observed in the production of all the evaluated collagen types (COL-I, COL-III, COL-IV, and COL-VI) (*p *< 0.001), as well as in elastin (*p* < 0.001) and TGF-β1 (*p *< 0.01), compared to the control (Fig. 6A–E), reflecting its potent pro-collagen and regenerative effect. Notably, HA + DX filler demonstrated a pronounced effect on COL-III production, with levels significantly higher (*p *< 0.001) than those observed in both the control and other treatments (Fig. 6B). This strong stimulation highlights its specific advantage in promoting COL-III, a critical component for skin repair and elasticity.

HA + CaHa also showed significant differences in COL-I (Fig. 6A) (*p *< 0.001), COL-III (Fig. 6B) (*p *< 0.01), and TGF-β1 (Fig. 6F) (*p *< 0.01) production compared to the control. The results also indicated increased elastin production (Fig. 6E) (*p *< 0.001). The statistical analysis provided valuable insights into the performance of HA + DX filler compared to HA + CaHa on several parameters. When comparing these fillers, most parameters showed no statistically significant differences (*p*-values between 0.423 and 0.552). However, for COL-III, HA + DX exhibited significantly greater stimulation (*p *< 0.001), underscoring its distinct advantage in this specific area while performing overall similarly to HA + CaHa (Fig. 6B). In summary, these findings suggest that HA + DX filler shows a clear benefit over CaHa and PLLA in several key areas, with its superior effect on COL-III standing out prominently.

Interestingly, the observations with the remaining fillers, CaHa and PLLA, revealed that CaHa treatment was associated with a significant increase in COL-I (Fig. 6A) (*p *< 0.05), COL-III (Supplementary 2B) (*p *< 0.05), and TGF-β1 (Fig. 6F) (*p *< 0.001) production compared to control, although its effect on COL-IV, COL-VI, and elastin (Supplementary 2C, D, and E) was lower than the above-mentioned treatments. On the other hand, PLLA showed a significant increase in COL-I (*p *< 0.01), COL-III (*p *< 0.05), COL-VI (*p *< 0.05), and TGF-β1 (*p *< 0.001) compared to control (Supplementary 2A and F). In the case of COL-IV and elastin, PLLA demonstrated a more limited effect (Supplementary 2B, C, D and E), with no differences compared to the control (*p *> 0.05), indicating a more modest impact on the stimulation of these components. Looking at the comparison between HA + DX filler and CaHa filler revealed significant differences in all parameters, with HA + DX filler consistently demonstrating a stronger effect, particularly in COL-III production (*p *< 0.001) compared to CaHa filler (Supplementary 2). The comparison between HA + DX filler and PLLA filler yielded more variable results, with statistically significant differences observed in COL-III, COL-IV, and elastin (*p *< 0.001) (Supplementary 2).

These results reinforce that HA + DX filler exhibits the strongest effect among the treatments evaluated, while the other products, though effective, show varying capacities for inducing different types of collagen and other components, with their impact depending on the specific collagen or elastin evaluated.

## Discussion

Recent studies suggest that dermal fillers may enhance skin regeneration by stimulating collagen and elastin production, promoting fibroblast migration, and improving skin texture and elasticity, beyond their primary role in volume correction [15]. In particular, it has been demonstrated that certain HA-based fillers, such as Hylan Gel Dermal Filler DX, which combines HA with dextranomer microspheres, not only provide volumetric enhancement but also induce higher production of type I and type III collagen in human fibroblasts [14, 16–19]. This suggests that this dermal filler may have a more active role in restoring skin architecture, extending beyond merely filling dermal spaces. However, this information has been largely absent from the current literature. In this context, our study compared Hylan Gel Dermal Filler DX (HA + DX), which includes dextranomer in its formulation, with other commercially available fillers such as Genefill Soft Fill (HA), Harmonyca® (HA + CaHa), Radiesse® (CaHa), and Sculptra® (PLLA), to evaluate its effects on cellular hydration, fibroblast migration, collagen and elastin production, and its influence on pro- and anti-inflammatory cytokines. These characteristics are crucial not only for facial aesthetics but also for regenerative applications that require effective fibroblast activation and sustain structural improvement over time.

Different HA fillers exhibit varying physical properties, including concentration, gel-to-fluid ratio, and degree of cross-linking, which can affect their performance and patient outcomes [20]. The effectiveness of dermal fillers depends on both the quality of the product and the practitioner's skill in application [21].

The physicochemical of the HA + DX filler align with physiological skin conditions, ensuring compatibility and reducing the risk of irritation or adverse reactions (Table 1) [22]. The observed turbidity of 23% reflects the presence of dextranomer particles, which contribute to the filler’s opalescent appearance [23] and the reduced probability of the Tyndall effect [24]. The swelling factor (121%) indicates a high capacity for water retention, a crucial property for dermal fillers as it ensures prolonged hydration and volumizing effects in treated areas. Dermal fillers with high water retention have been shown to improve both the longevity and natural appearance of the results, an important consideration in aesthetic treatments [25]. Furthermore, the osmolality of 426 mOsmol/kg falls within an optimal range for maintaining tissue hydration without causing cellular damage or tissue dehydration, which is essential for minimizing complications such as edema [26].

Rheologically, the HA + DX filler demonstrated a predominantly elastic profile at higher frequencies, with a G' of 4206.3 Pa at 10 Hz (Table 2). This suggests strong structural integrity, a key property for maintaining volume in areas requiring support, such as the cheeks or chin. High storage modulus (G') values are associated with fillers that offer long-lasting structural support [20]. In contrast, at lower frequencies (1.6 Hz), the filler displayed more viscous behavior, with a tan delta of 1.2, which allows for better adaptability in dynamic areas of the face, such as around the mouth and eyes. This balance between elasticity and viscosity is critical for aesthetic procedures, ensuring that the filler maintains both stability and flexibility under different stress conditions [27].

HA molecules, with their negatively charged subunits, excel at attracting and retaining water, supporting the extracellular matrix's structure, and maintaining cellular hydration, such as in fibroblasts. By preventing rapid water loss, HA helps sustain long-term cellular hydration, which is essential for maintaining healthy tissue function, promoting wound repair, and enhancing cell signaling in the extracellular matrix [23]. The analysis of hydration in NHDF and J774A.1 cells revealed that both HA and HA + DX fillers significantly increased cellular water content compared to the control (*p *< 0.05) (Table 3). However, the HA filler used in this study exhibited a more pronounced hydrating effect within the first 24 hours, making it ideal for treatments requiring rapid improvement in skin hydration. The HA + DX filler demonstrated superior long-term hydration maintenance, suggesting it is more suitable for treatments aiming for sustained and durable hydration. Comparing these fillers with other dermal fillers with different compositions, CaHa and HA + CaHa were noted for their early hydration capabilities (Table 4). Conversely, in this study, PLLA showed high hydration as described in some literature studies [28], however the least pronounced effect in terms of hydration in comparison with the other products tested. These findings are consistent with previous studies highlighting HA as a potent hydrating agent due to its ability to retain water in the extracellular matrix [29, 30]. Although HA + DX was less effective in initial hydration compared to CaHa and HA + CaHa, it stands out for its sustained hydration effect, which could be attributed to prolonged stimulation of collagen production and tissue remodelling. These findings suggest that Hylan Gel Dermal Filler DX not only provide immediate volume but also stimulates long-term improvements in skin quality through extracellular matrix production.

Several studies have highlighted the importance of biomechanical factors in cell migration and wound healing [31, 32]. Fibroblasts migrate together to establish deformation gradients that guide their movement, with optimal migration occurring on substrates with specific mechanical properties [24]. Novel dermal fillers have shown superior efficacy in promoting cell migration and collagen synthesis compared to traditional HA fillers [4]. The study of cell migration revealed that HA + DX filler significantly outperforms HA filler in stimulating fibroblast migration. At 72 hours, HA + DX filler achieved complete wound closure in NHDF cells, while HA filler exhibited a more gradual and less efficient effect (Fig. 1). This observation suggests that the components of HA + DX filler may activate key molecular pathways involved in cell migration, such as signaling through TGF-β1 and other cytokines associated with tissue repair and remodeling [33]. In comparison, CaHa and PLLA showed more moderate effects on fibroblast migration, with PLLA being the least effective among the fillers evaluated (Fig. 2). HA + DX's ability to enhance cell migration highlights its potential for promoting true regenerative healing rather than scar formation, offering a clinical advantage in wound healing and tissue regeneration, especially in dermal rejuvenation and post-trauma treatments [34].

Cross-linked HA injections were shown to increase collagen synthesis in photodamaged skin, potentially through mechanical stretching of dermal fibroblasts [1]. In vitro experiments with human dermal fibroblasts revealed that different HA-based fillers elicit varying responses in the production of extracellular matrix components, with some fillers showing superior stimulation of type I and III collagen and elastin [3]. HA + DX demonstrated a notably superior ability to stimulate the production of various collagen types (COL-I, COL-III, COL-IV, COL-VI) and elastin compared to HA (Fig. 2A–D). This broad-spectrum collagen-inducing capacity suggests a significant advantage in addressing age-related skin deterioration, such as loss of elasticity, texture, and structural integrity [35]. Type I and type III collagen, in particular, are critical for skin repair and elasticity, essential for maintaining youthful skin texture and function^26^. The stimulation of elastin production by HA + DX further strengthens its role in restoring skin resilience and preventing dermal aging (Fig. 2E). Elastin, often underappreciated, plays a key role in maintaining skin elasticity, and its depletion is a hallmark of aging skin [36]. Therefore, the ability of HA + DX to stimulate both collagen and elastin synthesis positions it as a comprehensive regenerative agent. Compared to other fillers, such as HA + CaHa and CaHa, both demonstrated significant effects on collagen production, particularly on COL-I and COL-III (Fig. 6A and B; Supplementary 2A and B). However, their ability to induce COL-IV and COL-VI was less pronounced (Fig. 6C and D; Supplementary 2C and D), highlighting a potential limitation in addressing more complex extracellular matrix remodeling. Similarly, PLLA proved effective in promoting type I collagen production but showed a limited impact on other collagen types and elastin, aligning with previous clinical reports on its long-term collagen-inducing effects. Despite the effectiveness of CaHa and PLLA in certain aspects of collagen stimulation, the more robust and broad-spectrum effect of HA + DX suggests a superior capacity for comprehensive tissue regeneration and the potential of this dermal filler to enhance skin quality through collagen induction.

Cross-linked HA dermal fillers can stimulate collagen synthesis in photodamaged skin by mechanically stretching fibroblasts, with different HA-based fillers eliciting varying fibroblast responses and influencing extracellular matrix production and TGF-β levels (3). The formulation of dermal fillers can significantly influence their immunomodulatory properties, with gel bioscaffolds promoting anti-inflammatory monocyte polarization and enhanced tissue regeneration compared to filler particles [37]. The cytokine modulation profile observed in HA + DX -treated fibroblasts and monocytes further supports its enhanced regenerative potential (Figs. 3 and 4). HA+ DX filler significantly increased TGF-β1 (Figs. 3A and 4A) and IL-10 (Figs. 3D and 4D) levels while reducing TNF-α (Fig. 4B), a profile conducive to anti-inflammatory and pro-regenerative processes. TGF-β1, in particular, plays a pivotal role in collagen production and fibroblast proliferation, acting as a key molecular signal for tissue regeneration and extracellular matrix synthesis [38, 39]. This elevation of TGF-β1 (Fig. 4A) with HA+ DX treatment suggests that its molecular mechanism of action likely involves the activation of pro-collagen signaling pathways, which may explain its superior performance in collagen and elastin synthesis. The modulation of IL-10 (Fig. 4D), an anti-inflammatory cytokine, further suggests that HA + DX may create a favorable environment for wound healing and tissue remodeling by mitigating chronic inflammation, which is often detrimental to long-term skin regeneration [40, 41]. In contrast, HA filler exhibited a less pronounced modulation of cytokine production, correlating with its lower regenerative effects compared to HA+ DX. Similarly, although HA + CaHa and CaHa promoted a cytokine profile that supports tissue regeneration, their impact on TGF-β1 (Supplementary 2F) was less significant, which may limit their potential for broader regenerative applications. PLLA showed limited cytokine modulation (Supplementary 2F) consistent with its lower efficacy in stimulating fibroblast migration and extracellular matrix production.

One important consideration is the elevation in IL-6 levels observed with HA + DX (Fig. 4E). Recent studies have indicated its role in collagen synthesis and fibroblast activation [42]. Moreover, in controlled amounts, IL-6 may play a pivotal role in enhancing the regenerative processes by fostering a localized, transient inflammatory response that stimulates wound healing and matrix remodeling [43–45]. Therefore, the observed IL-6 elevation may contribute to the enhanced collagen and elastin production [46, 47] observed with HA+ DX filler, potentially supporting its tissue-regenerative capacity in a manner that complements its other cytokine effects.

The findings of this study indicate that HA + DX offers a more comprehensive and robust approach to facial rejuvenation, outperforming other fillers in terms of collagen and elastin production as well as cytokine modulation. Its ability to simultaneously stimulate key extracellular matrix components and regulate pro-regenerative cytokines underscores its potential for advanced tissue regeneration and rejuvenation therapies.

The implications of these findings are substantial and far-reaching. HA + DX emerges as a frontrunner in the field of dermal fillers, offering not only prolonged hydration but also significant tissue regeneration benefits. Its superior ability to enhance collagen and elastin production, coupled with its influence on cytokine modulation and fibroblast migration, positions it as a highly effective treatment for skin rejuvenation, wound healing, and post-traumatic regeneration. The dual-action profile of HA + DX, providing both immediate hydration and long-term regenerative effects, sets it apart from other fillers. This combination suggests that Hylan Gel Dermal Filler DX could offer a more comprehensive and sustainable solution for improving skin quality, potentially reducing the need for frequent treatments and enhancing overall patient satisfaction. Moreover, the study indicates that clinicians may be able to personalize treatment regimens by combining Hylan Gel Dermal Filler DX with other fillers, tailored to the individual needs of patients and the anticipated clinical response time. This approach could optimize therapeutic outcomes and provide a more customized treatment experience.

A limitation of this study is that while hybrid products (HA + DX and HA+CaHa) are ready-to-use formulations, CaHa and PLLA products require dilution before use. Due to practical constraints, it was not possible to test multiple dilution ratios for CaHa and PLLA; therefore, only one dilution was tested, specifically at a concentration of 1:2:1 for PLLA (Sculptra) and 1:2.75 for CaHa (Radiesse). This limitation is noteworthy, as different dilution ratios can significantly affect the rheological properties, particle distribution, and, ultimately, the product's bio-stimulatory effect and efficacy, as highlighted in existing literature [48–51]. Studies have shown that CaHa, in particular, may exhibit variable bio-stimulatory outcomes based on the degree of dilution, influencing cell interaction and collagen production rates [52]. Similarly, the particle size and spread of PLLA can change with dilution, which may impact vitro cell responses and migration patterns [51, 53]. Future studies should explore a range of clinically relevant dilutions to more fully capture these potential variations and provide a more comprehensive comparison. Another limitation is the in vitro nature of this study. In vitro experiments do not fully replicate the complex biological environment of in vivo conditions, which can influence the behavior and efficacy of the fillers. Therefore, the results should be interpreted with caution, and further in vivo studies are needed to validate the findings.

In summary, Hylan Gel Dermal Filler DX demonstrates, in vitro, significant versatility and efficacy in both aesthetic and regenerative applications. Its ability to deliver immediate hydration while promoting structural improvements makes it a valuable tool in dermatologic practice. Future research could further investigate its use in combination with other therapeutic agents, aiming to maximize clinical benefits across various medical contexts.

## Supplementary Information

Below is the link to the electronic supplementary material.

Supplementary file1 (DOCX 672 kb)

## Data Availability

Following Springer Nature’s Data Policy Type 3, all relevant raw data described in this study are available upon reasonable request from the corresponding author, Alejandro Escamilla-Sánchez. The data will be shared exclusively for non-commercial purposes while ensuring participant confidentiality. The data supporting the findings of this study are available from the corresponding author, Alejandro Escamilla-Sánchez, upon reasonable request (jandromilla@uma.es). Due to privacy and ethical restrictions, individual patient data are not publicly accessible.
