# Supplementary material for: Enhanced Facial Rejuvenation: Biostimulatory Effects of Hylan Gel Dermal Filler DX on Collagen Synthesis and Tissue Regeneration
Source: Aesthetic Plast Surg. 2025 Oct 13;50(2):707–25. doi: 10.1007/s00266-025-05245-5 (PMC12957018; doi:10.1007/s00266-025-05245-5)
Supplement: Supplementary file 1 — Supplementary file1 (DOCX 672 kb) [file 266_2025_5245_MOESM1_ESM.docx]

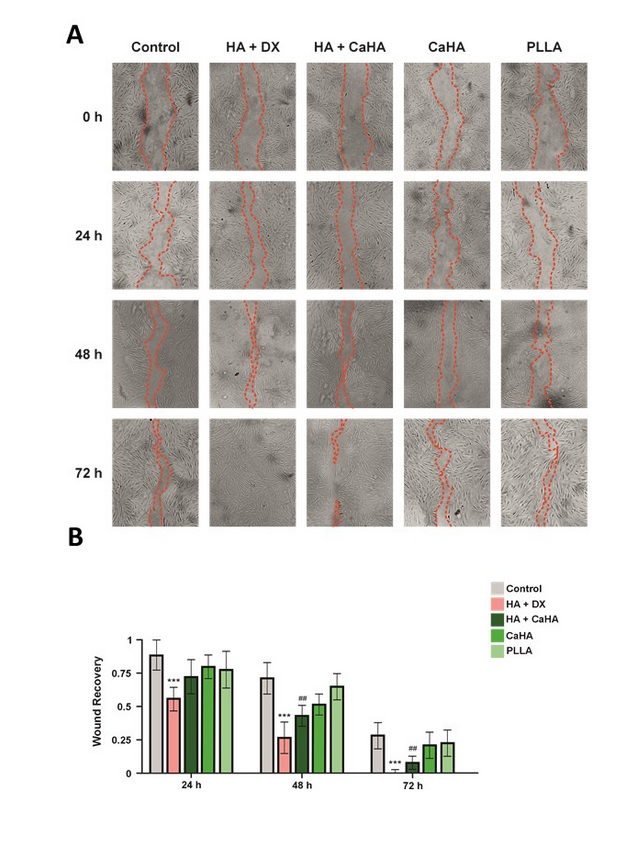


**Supplementary 1. Comparison of cell migration Among Dermal Fillers.** (A) Microscopic images illustrating fibroblast migration after 24, 48 and 72 hours of treatment with control, HA + DX, HA + CaHa, CaHa and PLLA fillers. HA + DX filler demonstrated the highest level of migration, followed by HA + CaHa, with CaHa and PLLA showing more moderate effects. (B) Quantitative analysis of cell migration across the treatments. HA + DX filler significantly enhanced fibroblast migration compared to the other fillers indicating its superior efficacy in promoting cell movement critical for tissue repair. Data are presented as mean ± SEM; significant differences from the control are indicated with asterisks (*) for HA + DX filler, where ^*^p < 0.05, ^**^p < 0.01, and ^***^p < 0.001; with hash (#) for HA + CaHa, where ^#^p < 0.05, ^##^p < 0.01, and ^###^p < 0.001; with et (&) for CaHa, where ^&^p < 0.05, ^&&^p < 0.01, and ^&&&^p < 0.001, and with dagger (†) for PLLA, where ^†^p < 0.05, ^††^p < 0.01, and ^†††^p < 0.001.


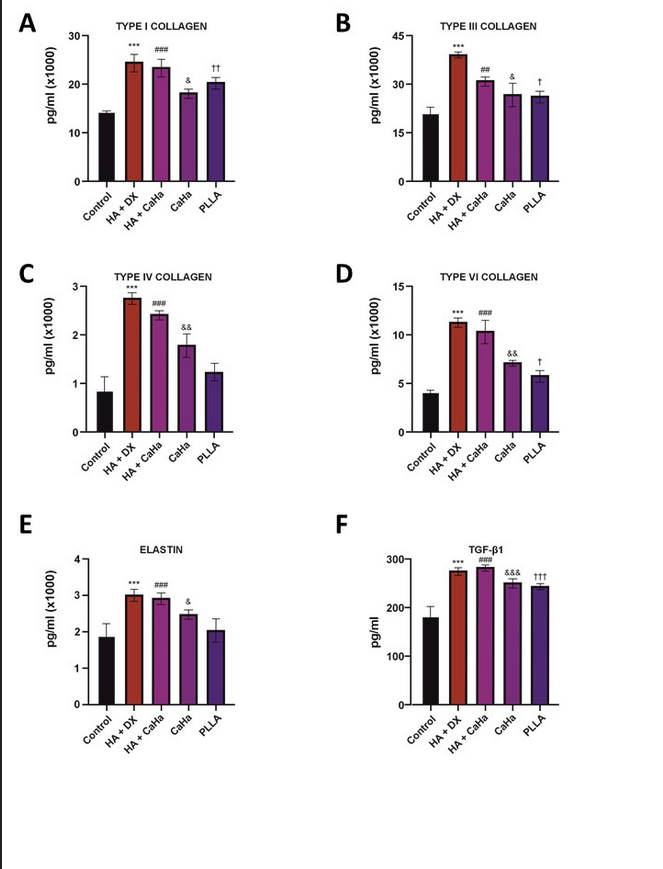


**Supplementary 2. Comparison of collagen and TGF-β1 production in a co-culture system among dermal fillers.** (A-F) Levels of collagen (COL-I, COL-III, COL-IV, COL-VI), elastin, and TGF-β1 measured in fibroblast supernatants after 24-hour treatment with HA + DX filler, HA + CaHa, CaHa, PLLA, and an untreated control. HA + DX filler significantly increased the production of all evaluated collagen types, elastin, and TGF-β1, outperforming other treatments. HA + CaHa and CaHa also demonstrated notable effects, particularly on COL-I, COL-III, and TGF-β1, while PLLA showed a more modest impact on collagen and elastin synthesis. Data are presented as mean ± SEM; significant differences from the control are indicated with asterisks (*) for HA + DX filler, where ^*^p < 0.05, ^**^p < 0.01, and ^***^p < 0.001; with hash (#) for HA + CaHa, where ^#^p < 0.05, ^##^p < 0.01, and ^###^p < 0.001; with et (&) for CaHa, where ^&^p < 0.05, ^&&^p < 0.01, and ^&&&^p < 0.001, and with dagger (†) for PLLA, where ^†^p < 0.05, ^††^p < 0.01, and ^†††^p < 0.001.
